# Positive-Strand RNA Viral RdRps: From Structure Conservation and Activity Assay to Drug Development

**DOI:** 10.3390/molecules31122044

**Published:** 2026-06-11

**Authors:** Siyuan Zhao, Ziyu Lin, Minglian Wang

**Affiliations:** College of Chemistry and Life Science, Beijing University of Technology, Beijing 100124, China; zhaosiyuan@emails.bjut.edu.cn (S.Z.); linziyuoptfreq@163.com (Z.L.)

**Keywords:** positive-stranded RNA virus, RNA-dependent RNA polymerase (RdRp), RdRp inhibitor, RdRp assay

## Abstract

Positive-strand RNA viruses represented by SARS-CoV-2 and DENV spread globally with high infectivity and frequent mutations, posing a severe threat to human health. However, specific and effective antiviral drugs remain limited. RNA-dependent RNA polymerase (RdRp) plays a critical role in the genome replication of RNA viruses and shows high structural conservation. No homologous protein exists in human cells, making RdRp an effective, safe, and broad-spectrum antiviral target. Therefore, establishing an RdRp activity assay for drug development based on its mechanism is of great significance. This paper summarizes the structural characteristics of RdRp and the methods for constructing RdRp activity assay, especially for cell-free activity assay. In addition, several reported RdRp inhibitors and their pharmaceutical progress are also reviewed, aiming to provide a reference for the development of novel antiviral drugs targeting RdRp.

## 1. Introduction

In recent years, Single-positive-stranded RNA (+ssRNA) viruses, which possess a mRNA-like coding genome, have posed serious threats to human health. Typical representatives include hepatitis C virus (HCV), Zika virus (ZIKV), dengue virus (DENV), chikungunya virus (CHIKV), and Severe acute respiratory syndrome coronavirus 2 (SARS-CoV-2) [1,2]. Encoded by viral genes, RNA-dependent RNA polymerase (RdRp) is a multi-domain nucleic acid polymerase present in all RNA viruses except the family Retroviridae [3]. Due to its lack of proofreading capability, RdRp exhibits an error rate approximately ten thousand times higher than that of DNA polymerases. This high error rate leads to frequent mutations, and enables fast and widespread virus transmission. However, effective drugs remain limited, so it is urgent to develop broad-spectrum antiviral drugs [4,5].

RdRp is structurally conserved and essential for viral RNA synthesis, making it a broad-spectrum antiviral target [1,6,7]. RNA synthesis in human cells relies on DNA-dependent polymerases, which share no homology with RdRp. This distinction makes RdRp also a safe target for antiviral drug development [8]. To screen antiviral candidate drugs targeting RdRp, it is essential to establish a robust RdRp activity assay. Since the genomic RNA of positive-strand RNA viruses is infectious, it may be translated, then packaged into viruses or virus-like particles after cell entry. However, many cell-based systems avoid this risk by using only subgenomic elements or replication-deficient viral components, which present low biosecurity risks. Cell-free systems, on the other hand, not only pose no biosafety risk but also can exclude interfering factors such as differences in cell vitality, endogenous metabolites, metal ions, and redox environments that affect RdRp. Thus, establishing a cell-free RdRp activity assay can ensure better safety and more reproducible results.

RdRp inhibitors can be broadly divided into nucleoside inhibitors (NIs) and non-nucleoside inhibitors (NNIs) based on their chemical structures. NNIs can be further divided into synthetic molecular drugs and natural products [9]. This paper analyzes the advantages of RdRp as a broad-spectrum antiviral drug target, summarizes key points for constructing an RdRp activity assay, and introduces several RdRp inhibitors along with their clinical progress.

## 2. Structural and Functional Characteristics of RdRp

### 2.1. Protein Structure of RdRp

**Figure 1:**
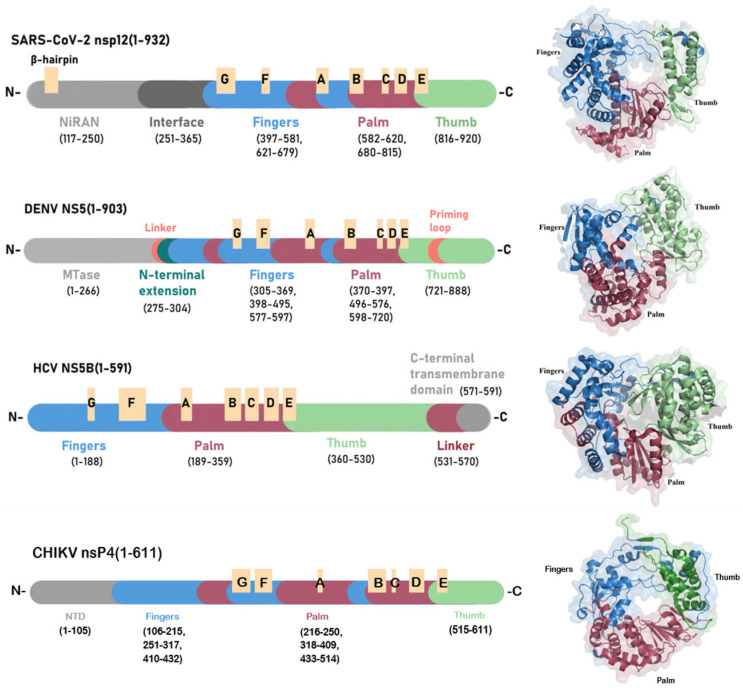
Linear and spatial structures of the RdRps in four representative viruses. The seven motifs 3.6.(A–G) in the RdRp active region of SARS-CoV-2, DENV, HCV and CHIKV are sequentially consistent, and the overall spatial conformations of the enzyme molecules formed are similar. The linear and spatial structures of the RdRps were shown and analyzed using PyMOL-3.1.6.1 software. All the primary structure data were derived from the PDB database, respectively SARS-CoV-2 nsp12 (PDB, 7BW4), DENV NS5 (PDB, 4V0Q), HCV NS5B (PDB, 3FQK) and CHIKV nsP4 (PDB, 7Y38).

The RdRp of +ssRNA viruses exhibits a classical right-handed shape composed of finger, thumb, and palm domains [3,10,11,12,13,14], performing functions including template binding, nucleotide triphosphate (NTP) recruitment, and RNA polymerization [12]. Figure 1 shows the linear and spatial structures of RdRp from SARS-CoV-2, DENV, HCV, and CHIKV. Their structure data were sourced from the PDB database: SARS-CoV-2 nsp12 (PDB, 7BW4), DENV NS5 (PDB, 4V0Q), HCV NS5B (PDB, 3FQK), and CHIKV nsP4 (PDB, 7Y38). The finger and thumb domains of RdRp are located at the N- and C-terminal of the sequence, forming the active site as well as the entry and exit channels for RNA synthesis. The finger domain channels NTPs into the active site, while the thumb domain spans the entire polymerase and permits the passage of the RNA chain [3]. Based on this structure, RdRp binds to viral genomic RNA, recognizes and recruits free cellular NTPs, and adds them to the 3′-hydroxyl terminal of the nascent RNA chain. RNA synthesis proceeds in the 5′→3′ direction to replicate viral genomes [3,9,15,16].

The active catalytic site of RdRp contains seven essential motifs, including motifs A–E in the palm domain and motifs F and G in the finger domain [3,10,12,14,17]. These motifs are conserved in both sequence and structure, arranged within the RdRp active site to coordinate reaction substrates and participate in nucleotide recognition and polymerization reactions with the template [8]. Although the linear sequences of these motifs differ among viral families, their spatial structures around the active site are highly similar, representing a crucial conservation feature of RdRp [18]. Among these motifs, A and C are typically conserved across most viral polymerases [8,10]. They contain the classic divalent cation-binding Asp residues, which participate in catalytic reactions by binding Mg^2+^ ions [3,10,18]. Motifs B, D, E, and G contribute to nucleotide recognition and coordination [17]. Motif B includes a highly conserved Ser-Gly sequence [18]. Motif D forms an antiparallel β-sheet with motif A. During the closure of the polymerase active site, it moves antiparallel to motif C relative to motif A, extending the β-sheet from two to four strands [18]. Motifs F and G are located in the finger subdomain, interacting with the template RNA and guiding it toward the active site [19,20]. Hydrophilic residues in motif F participate in trapping NTPs into the entry channel [10] and also interact with the primer RNA, thereby stabilizing the incoming nucleotide in the correct catalytic position [18,20]. Most RdRps possess positively charged pathways for the primer or template entry channel, NTP entry channel, and nascent chain exit channel, all converging into the central catalytic cavity. We propose that this positive charge enables the enzyme to interact with the negatively charged RNA template and the buffering system. Beyond these fundamental motifs, specific residues can also recognize the 2′-hydroxyl group of the ribose in NTPs, enabling strict synthesis of RNA rather than DNA [19].

### 2.2. Special Domains of RdRp

Although the RdRps share a highly similar spatial structure, some RdRps possess unique domains or cofactors at their N- or C-termini [3].

Within the family *Coronaviridae*, taking SARS-CoV-2 as an example, its RdRp core is non-structural proteins (nsp)12 of 932 amino acids, together with one subunit nsp7 of 87 amino acids and two nsp8, each of 197 amino acids [8,10,14]. In this complex, nsp12 is typically regarded as the RdRp molecule due to its central role in RNA synthesis. One nsp7 and one nsp8 bind to the thumb domain, while another nsp8 binds to the finger domain of nsp12 [10]. The extension process between nsp8 and the active site employs a pulley mechanism in which positively charged residues interact with the RNA backbone, facilitating sliding along the nascent RNA and preventing premature dissociation of RdRp during replication [1,19]. In addition, nsp8 also possesses primerase activity, which enhances the efficiency of RNA synthesis [21].

Within the family *Flaviviridae*, the NS5 protein of DENV (approximately 900 amino acids in total length) contains two major domains: the N-terminal methyltransferase (MTase) domain of 235 amino acids and the C-terminal RNA polymerase domain of 564 amino acids [22,23,24]. The MTase domain is tightly connected to the RNA polymerase domain, with each maintaining an independent conformation and performing distinct functions [11]. Because of the MTase domain, the NS5 protein can participate in both RNA replication and the RNA capping process [25].

Another representative member of the family *Flaviviridae* genus, HCV, possesses its RdRp functional domain within the NS5B protein [13]. HCV NS5B is also a tail-anchored protein. The RdRp catalytic domain, starting from the amino terminus, forms the main body of NS5B, while the carboxy terminus forms a transmembrane domain that anchors the protein to the membrane [13]. All non-structural proteins of HCV interact with the cell membrane, enabling viral replication to occur at the membrane surface and thereby reducing the dimensionality of the reaction [13].

Within the family *Togaviridae*, the N-terminal domain of CHIKV nsP4 is a genus-specific structure characterized by intrinsic disorder that requires binding to nsP1 for stabilization. It contains the conserved N-terminal Y1 residue, a helix-turn-helix motif, and an anti-parallel β strand [26]. This domain inserts into the RNA-binding groove and acts similarly to the priming loop of DENV NS5 RdRp. It also interacts with nsP1 and nsP2 to promote RNA binding and replication complex assembly [26].

Although diverse viral RdRps have evolved genus-specific N-/C-terminal domains and cofactor systems to accommodate their distinct replication modes, the core palm-finger-thumb domains responsible for RNA synthesis are highly conserved in evolution, which is a core basis for the development of broad-spectrum antiviral targets. Table 1 summarizes key findings from recent studies on RdRp variation and conservation, which are broadly categorized into structural comparisons of RdRp at the viral genus and strain levels.

Such conservation characteristics are more obvious among subtypes and mutant strains of the same virus. Studies have shown that the mutation frequency in the HCV NS5B RdRp domain is lower than in other non-structural proteins (NS3/4A and NS5A) [37]. Compared with the original strain, the Omicron variant of SARS-CoV-2 has approximately 60% of total mutations in the spike protein coding region. In contrast, mutations in RdRp only account for about 3.6% of total mutations [38,39]. Molecular alignment of RdRp protein models between the Omicron variant and the original strain showed that the RMSD values between the two were within 0.5–0.6 Å, indicating that the amino acid mutations had little impact on the overall structure of RdRp, confirming the conservation of RdRp across strains [32]. Similarly, a study on coxsackievirus B3 (CVB3) reported that the 3D^pol^ amino acid sequences among CVB1-6 share more than 95% identity, with key residues fully conserved across all six serotypes [40]. This trend is also observed in norovirus genogroup I (GI), where the RdRp gene shows 70.5–100% nucleotide identity and 81.2–100% amino acid identity across all P-genotypes [41].

Accumulated structural evidence has supported a critical evolutionary hypothesis: all viral RdRps originated from a common ancestor, and single-stranded RNA viral RdRps evolved from ancestral double-stranded RNA viral RdRps [31]. Despite limited sequence similarity among different viral species, the spatial structure of RdRps and the conserved motifs at their catalytic centers are consistently conserved. This provides a robust foundation for identifying RdRps as broad-spectrum antiviral drug targets [31].

### 2.3. Two Mechanisms by Which RdRp Exercises Its RNA Synthesis Function

**Figure 2:**
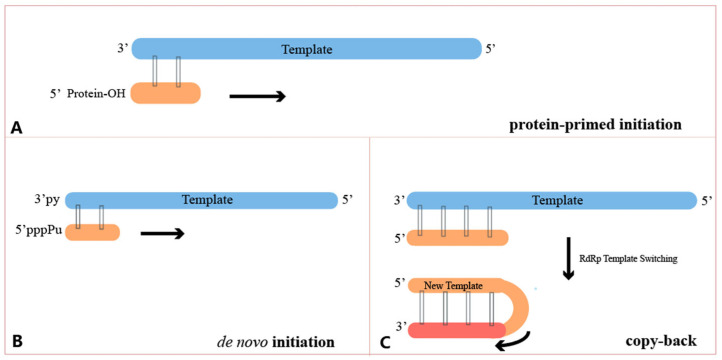
Protein-primed initiation and *de novo* initiation. Schematic description of RNA *de novo* mechanism and primer extension synthesis. (**A**) Protein-primed initiation uses a viral protein hydroxyl as primer. (**B**) *De novo* initiation synthesizes nascent RNA without a primer. (**C**) Copy-back mechanism generates a copy-back defective viral genome via RdRp template switching.

RdRp does not have the strict oligonucleotide primer dependence like DNA polymerase. Based on their primer dependency during RNA binding in cells, two main mechanisms have been broadly identified, as illustrated in Figure 2.

As shown in Figure 2A, protein-primed initiation refers to the mechanism in which the hydroxyl group of an amino acid residue on a protein molecule undergoes a condensation reaction with the first NTP to initiate RNA synthesis. This mechanism shows that RdRp can use not only the 3′-hydroxyl group of nucleotides as a primer but also other molecules, such as the hydroxyl group of amino acids, as “functional primers” to trigger the RNA primase reaction and start RNA synthesis. For example, the VPg protein of picornaviruses covalently binds to the 5′end of the viral genome, which contains a highly conserved cis-acting replication element (*cre*). RdRp uses *cre* as a template and the hydroxyl group of the conserved tyrosine residue in VPg as primer, adding two uridine monophosphates to form the VPgpUpU-OH intermediate, thereby initiating viral RNA replication. Similarly, the synthesis of poliovirus negative-strand RNA also begins with the hydroxyl group of a tyrosine residue on a protein serving as a primer.

*De novo* initiation refers to the process by which RNA polymerase synthesizes RNA without primer assistance, as depicted in Figure 2B. RdRp recognizes the initiating NTP by matching it to the 3′-terminal base of the template RNA through complementary base pairing. RNA synthesis starts with the formation of the first phosphodiester bond between the 3′OH of the initiating NTP and the 5′triphosphate of the second NTP [42]. The NS5 RdRp of the *Flavivirus* genus contains a primer loop (residues V785–D810) responsible for the allosteric positioning of the active site at the 3′terminus of the RNA template [13,27]. This loop enables the dinucleotide synthesis at the 3′end of the template, facilitating the *de novo* initiation of the RNA chain [18,43]. Figure 2C illustrates a related process known as the *de novo* copy-back mechanism. In this mechanism, the RdRp detaches from the template, restarts synthesis in the reverse direction on the end of the new RNA strand, and produces copy-back defective viral genomes (cbDVGs).

Though some research on the cell-free RdRp activity assay reported that SARS-CoV-2 RdRp showed oligonucleotide primer dependency, it only provided RNA chain extension gels in the presence of primers, lack of control reaction without primers [44]. In other cell-free studies, the RdRp activity assay for ZIKV, DENV, and HCV all supported successful RNA synthesis without added primers [11,45,46,47,48]. This indicates that certain RdRps are not strictly dependent on oligonucleotide primers, additional primers primarily enhance the efficiency of RNA synthesis. However, most cell-free activity assays for SARS-CoV-2 RdRp are performed with exogenous primers. To evaluate SARS-CoV-2 RdRp activity and primer specificity, I. Petushkov et al. (2022) performed RNA extension reactions using primers of different lengths and templates and showed that RdRp efficiency depends on primer length and template matching [8]. We consider that there is no strict boundary between *de novo* initiation and Protein-primed initiation. Beyond copy-back mechanisms, *de novo* initiation also cannot rule out the possibility of hydroxyl group initiation. RNA chain can be initiated wherever an accessible hydroxyl group is available. However, these conclusions still require further verification and refinement through more in-depth studies on the functional mechanisms of different viral RdRps.

Furthermore, although RdRps from different viruses exhibit broadly similar conformations, their interactions with other non-structural proteins vary significantly. For example, DENV NS5 can independently display RdRp activity, recognizing templates and synthesizing new RNA chains without other auxiliary proteins [11,45,46,47]; however, in SARS-CoV-2, nsp12 requires the help of nsp7 and nsp8 to function. Nsp8 has primase activity, it can synthesize short RNA primers to help RdRp extend the growing chain [49,50]. During RNA elongation, the RdRp binds to the 3′end of the primer at an active site composed of residues D618, D760, and D761. After contacting the 6bp double-stranded structure of the primer, it initiates the extension of the nascent RNA chain [51].

## 3. Screening of RdRp Inhibitors and Establishment of a Cell-Free RdRp Activity Assay

The conservation of RdRp and its central role in viral genome replication make it a critical broad-spectrum antiviral target. The development of antiviral drugs targeting RdRp generally follows the standard drug discovery process, which includes three phases: inhibitor discovery, in vitro and in vivo experiments, and clinical trials. Recent studies are mainly focused on developing inhibitors of SARS-CoV-2 RdRp. This section focuses on the discovery and validation of SARS-CoV-2 RdRp inhibitors as drug candidates.

### 3.1. Virtual Screening of RdRp Inhibitors

Currently, commercialized RdRp enzymes are scarce, and the few available products are expensive and exhibit low activity (product descriptions often state “activity unknown”). Laboratory expression of RdRp also faces challenges of low yield and expression difficulties. Therefore, potential RdRp inhibitors are first identified by computational and simulation screening, followed by in vitro enzymatic activity assays for experimental validation.

Virtual screening, including molecular docking and molecular dynamics simulations, has been widely applied in drug discovery [24]. A. Vijay et al. (2024) [52] established a novo strategy combined with well-tempered metadynamics for binding energy validation, without relying on standard docking tools for primary screening. Taking remdesivir as a reference, their work focused on key RdRp residues Asp760, Asp761, and Asn691. From 5375 compounds, they identified 7 *de novo* molecules and 3 repurposed drugs. T. Uengwetwanit et al. (2022) [28] use Genetic Optimization for Ligand Docking (GOLD) software with the Astex Potential (ASP) scoring function, which adopts a genetic algorithm to explore ligand conformations. They screened compounds from the ZINC ChemDiv database targeting the conserved catalytic pocket of SARS-CoV-2 RdRp and screened 10 candidates based on the binding affinity and interactions with conserved residues. Bazzi-Allahri et al. (2024) [53] constructed a quantitative structure-activity relationship (QSAR) classification model based on the Simplified Molecular Input Line Entry System (SMILES) using 2377 compounds. They further performed virtual screening of 60.2 million compounds from the ZINC, ChEMBL, Molport, and MCULE databases using pharmacophore (PH4) and QSAR models targeting the active site of SARS-CoV-2 RdRp [53]. Unlike structure-based docking, this method relies on ligand-structure features and is suitable for large library screening. X. Du et al. (2024) [54] proposed a “ring-opening” modification strategy of natural nucleosides for antiviral design. By docking these analogues into the active site of RdRp, they confirmed that the “ring-opened” structure of guanosine exhibits broad-spectrum antiviral activity, highlighting a structure-based nucleoside optimization approach distinct from small-molecule library docking.

In summary, the above studies employ diverse computational strategies including *de novo* design, molecular docking, ligand-based virtual screening, and nucleoside structural optimization to discover small-molecule inhibitors binding to the conserved active site of SARS-CoV-2 RdRp. Since RdRp serves as the core enzyme catalyzing viral genomic RNA synthesis, these inhibitors work best when given at the early stage of infection to suppress viral replication, but will become far less effective in middle or late infection when virus levels rise rapidly. For disease prevention, RdRp has identical sequences in many coronaviruses according to previous studies. Bazzi-Allahri’s ADMET tests also prove the candidate drugs have good drug properties and can be easily absorbed in the gut. Therefore, high-risk people can take these medicines before virus exposure. The drugs will block the active site of RdRp and stop viruses from replicating right after entering cells, so they have great potential to be developed as preventive drugs.

### 3.2. RdRp Activity Assay

After virtual screening, experimental validation is required. The first step is to verify their RdRp inhibitory effects by in vitro and in vivo experiments. So, an effective RdRp activity assay is critical [24]. RdRp activity assays are categorized into two models: cell-based and cell-free. A cell-based RdRp activity assay works by expressing viral RdRp and specific reporter plasmids in living cells. The specific reporter plasmids generally contain viral *cre*, transcription termination elements, and reporter genes, which can only express luminescence after RdRp completes RNA replication and transcription, thereby reflecting enzyme activity [55,56,57]. A cell-free RdRp activity assay includes recombinant RdRp, external RNA templates and primers, as well as reaction buffer [11,45,58]. Compared with the cell-based system, cell-free systems avoid many confounding factors such as cell type, viability, plasmid transfection efficiency, and RdRp expression levels, offering the advantage of high reproducibility. Such systems have been widely used in current studies. This paper summarizes the technical methods for establishing cell-based and cell-free RdRp assay systems across multiple studies, as shown in Table 2 and Table 3.

#### 3.2.1. Expression of RdRp

**Figure 3:**
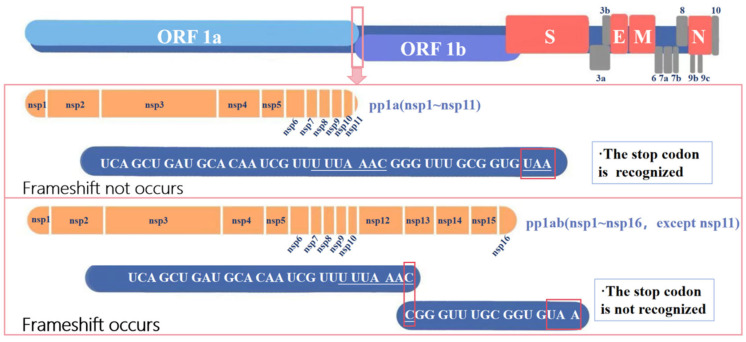
Programmed ribosome frameshift in SARS-CoV-2 genome. The slippery sequence “UUUAAAC” is located at nucleotides 21 to 27 after the nsp10 gene in the SARS-CoV-2 genome. Only when −1 PRF occurs can we translate the entire polyprotein 1ab, which contains nsp12 (RdRp) and downstream non-structural proteins. The slippery sequence was translated into partial amino acids of RdRp, without nsp11 production.

Constructing an active RdRp is the core for establishing the reaction system. Due to the large size of the RdRp molecule, it is challenging to obtain a full-length active recombinant RdRp. Although prokaryotic expression systems often produce protein inclusion bodies, which may impair expression efficiency and enzymatic activity, most studies still use prokaryotic expression as the main strategy, followed by purification using protein tags [11,45,46,50,64,66,67,68,69,75]. In contrast, eukaryotic expression plasmids are only constructed in cell-based and a few cell-free reaction systems [56,57,58,76]. In cell-based assays, RdRp is expressed inside living cells to support viral RNA replication in a physiologically relevant context. Unlike cell-free systems, where purified RdRp often maintains basal activity under optimized conditions, many viral RdRps require auxiliary proteins for proper folding and function inside cells [55,56,59,62]. We speculate that there are mainly two reasons for the limited applications of eukaryotic expression: first, recombinant proteins may cause toxic effects on host cells, leading to degradation by host proteases [45,50]. Second, when constructing eukaryotic reaction systems, retaining the programmed ribosomal frameshifting (PRF) sequence from viral genes can induce ribosomal slippage, directly reducing protein expression efficiency. The ribosomal frameshifting phenomenon in the SARS-CoV-2 genome is shown in Figure 3.

PRF is a mechanism used by some viruses to maximize the utilization of their genetic information during replication. Viral RNAs contain programmed frameshift signals that cause ribosomes to pause during protein translation. Most ribosomes resume translation in the original reading frame, while the others slip along the RNA and continue translation in a new reading frame. The SARS-CoV-2 genome is approximately 29.9 kb in length [77], with 16 non-structural proteins encoded by open reading frames (ORFs) 1a and 1b. The genes of structural proteins and cofactors are distributed in the subsequent smaller ORFs. SARS-CoV-2 nsp12 has RdRp activity and is encoded by ORF1b. During the translation of the SARS-CoV-2 genome, a unique slippery sequence in its mRNA and a subsequent frameshift-stimulating pseudoknot cause the ribosome to shift one nucleotide toward the 5′end during “reading frame” translation, known as the −1 PRF [78,79,80].

The core sequence “UUUAAAC” of the −1 PRF is located at nucleotides 21 to 27 downstream of the nsp10 gene. During translation, if a −1 PRF occurs, the ribosome shifts −1 frameshift along the mRNA, leading to the translation of the entire polyprotein 1ab (pp1ab). This product contains nsp12 and all downstream non-structural proteins. If −1 PRF does not occur, the ribosome translates in the original reading frame and only synthesizes the polyprotein 1a. Translation terminates at nsp11, preventing the translation of downstream non-structural proteins [78]. Studies indicate that the probability of −1 PRF is approximately 20% [81]. This low efficiency results in RdRp expression levels being less than 20% of its upstream proteins. Therefore, the slippery sequence must be removed by codon optimization [55] or disrupt the RNA secondary structure responsible for inducing ribosomal frameshifting [58,59,76]. Other groups also use PCR amplification with specifically designed primers to directly amplify an RdRp coding region without the slippery sequences [50,64,82].

Beyond the factors discussed, multiple non-structural proteins also take part in viral RNA replication [83]. For example, SARS-CoV-2 nsp13 has ATP-dependent 5′ to 3′ RNA helicase activity, which helps form complex secondary and tertiary structures in nascent RNA [51]. Meanwhile, nsp9, nsp10, nsp14, and nsp16 have been shown to regulate RNA 5′end cap synthesis [51]. The NS2A protein of HCV also participates in regulating RNA replication and viral assembly processes, which functions as a helicase [23].

#### 3.2.2. RNA Template and Primer Design

For laboratory safety, we do not use the original viral genome. Instead, a template RNA sequence is designed for the cell-free assays. It must contain a UTR sequence specifically recognized by the polymerase, typically 6 nt in length. The rest of the sequence has no specific requirements. Except when constructing copy-back self-priming templates (see also 2.3), secondary structures should be minimized. In addition, to improve the activity or reaction efficiency of recombinant RdRp, the template RNA strand should be significantly shorter than the viral genome length. Common lengths range from 20 to 500 nt [32,84].

SARS-CoV-2 RdRp exhibits primer dependence, so template design usually includes primers or self-priming templates (see also 2.4) [50,58,64,66,67,68,69,82]. In contrast, RdRps with *de novo* synthesis ability, such as those in the genus *Flavivirus*, generally do not require primers in the reaction system [11,45,46,75]. Furthermore, template sequences are adjusted to match the detection methods in different reaction systems. For instance, systems that use labeled NTPs for detection will add polyadenine sequences to templates to promote the incorporation of labeled NTPs into RNA products, thereby amplifying signals [45,46,50,73]. X. Bai’s team added biotin to the self-priming template to facilitate product detection [58]. Other groups have also added labels at the primers [66].

In cell-based systems, RNA template is not directly introduced into cells. Instead, a DNA plasmid encoding the viral minigenome is transfected into host cells [55,59,60,61,62,63]. The plasmid is first transcribed into RNA, and then recognized by viral RdRp as a template. Cell-based assays generally do not require exogenous primers. This is because the viral UTRs contain natural replication signals that recruit the RdRp together with host factors to initiate synthesis through either protein priming or *de novo* initiation.

#### 3.2.3. Method of RNA Detection

For cell-free reaction systems, denaturing RNA gel electrophoresis is the most common method for detecting RNA chain formation [11,50,58,64,66,68,69,82]. However, RNA is prone to degradation, so RNase inhibitors are typically added to the system, and exposure to the environment should be minimized. For longer templates, qPCR is also a standard detection method. Beyond electrophoresis and qPCR, fluorescence detection is another commonly employed method. It can be achieved in three ways: labeling primers or templates, incorporating fluorescently modified NTPs into nascent RNA strands, or using fluorescent dyes that bind to RNA duplexes or secondary structures [66]. The most commonly used approach is the incorporation of fluorescent NTPs. For instance, BBT-ATP ((2′-[2-benzothiazoyl]-6′-hydroxybenzothiazole) conjugated adenosine triphosphate) was used instead of ATP and incorporated into the RNA chain, with one molecule of BBTppi released per BBT ATP incorporated. After the RNA synthesis reaction, alkaline phosphatase hydrolyzes BBTppi into BBT and two phosphate groups, causing the system to fluoresce. Higher RdRp activity correlates with increased fluorescence intensity [46,75]. Additionally, studies have used radiolabeling assays to detect RdRp activity. They incorporate radioactive NTP into the reaction system or add radioactive tags, then measure the intensity of radioactive signals using methods such as liquid scintillation counting [11,45,50,64,82]. A. Shannon et al. (2020) further developed this method to calculate NTP incorporation rates based on the radioactive label [67].

For cell-based reaction systems, the complex composition—with different types and sizes of nucleic acids and nucleases coexisting—makes gel electrophoresis and qPCR detection challenging. Thus, fluorescence detection has become the most widely used method for these systems. J. Zhao et al. (2021) [59,75] constructed a Gauss luciferase reporter gene expression plasmid. They co-transfected cells with an RdRp expression plasmid and this reporter plasmid. RdRp recognized and replicated the mRNA containing the luciferase gene, thereby raising luciferase protein expression levels, which reflected the activity of RdRp. J.S. Min et al. (2021) [55] established a bioluminescence-based assay consisting of an RdRp expression plasmid and an active reporter plasmid carrying two fluorescent tags. Similarly, RdRp activity is reflected by analyzing the fluorescence intensity in the system. The results from the two fluorescence intensities are consistent, offering greater reliability than single fluorescence. Porcine epidemic diarrhea virus (PEDV) is a member of the Coronavirus genus. Uengwetwanit et al. (2022) [28] infected Vero cells stably expressing the eGFP fluorescent protein with mCherry-PEDV and tested several candidate compounds that target RdRp. The activity of the test compounds was ultimately evaluated by quantifying mCherry fluorescence intensity. However, due to the ease of fluorescence quenching, strict requirements for reaction timing, and complex composition of intracellular reaction systems, it is difficult to achieve consistent RdRp expression levels across different cell groups. Therefore, numerous technical details require optimization.

## 4. Current Status of RdRp Inhibitors’ Development

**Figure 4:**
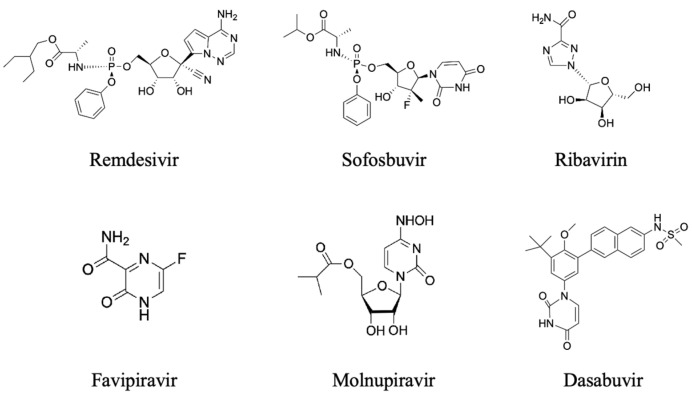
Molecular structure of RdRp inhibitors. The Molecular structure of six RdRp inhibitors, including Remdesivir, Sofosbuvir, Ribavirin, Favipiravir, Molnupiravir, and Dasabuvir.

RdRp inhibitors are generally divided into nucleoside inhibitors (NIs) and non-nucleoside inhibitors (NNIs) based on their chemical properties. Nucleoside RdRp inhibitors are misrecognized by viral RdRp and incorporated into the nascent viral RNA chain, thereby blocking viral RNA synthesis [9]. Non-nucleoside inhibitors are non-competitive inhibitors that bind to the allosteric site of RdRp, altering its conformation and thereby preventing viral RNA binding and elongation [3,85]. A number of effective RdRp inhibitors already exist. Meanwhile, reusing existing drugs is another practical way to develop new antiviral drugs and research targets [2]. Due to the structural conservatism of RdRp, existing antiviral drugs targeting RdRp, such as ribavirin, sofosbuvir, and remdesivir, are considered potentially applicable as broad-spectrum antivirals targeting RdRp [86]. This section briefly introduces six representative RdRp inhibitors that have been approved for marketing by the National Medical Products Administration (NMPA) or the U.S. Food and Drug Administration (FDA), along with several candidate RdRp inhibitor compounds. Figure 4 displays the chemical structures of these agents, while Table 4 lists their basic profiles, such as targeted viruses and nucleoside analogue characteristics.

### 4.1. Six Targeted RdRp Drugs Approved for Market

Ribavirin is the first synthetic nucleoside with broad-spectrum antiviral activity and was initially used in combination with interferon for treating HCV infection in 1998 [88]. Ribavirin is converted to ribavirin triphosphate (RTP) inside the cells [88], which induces the accumulation of mismatched nucleotides and thereby inhibits viral activity. Ribavirin primarily targets HCV NS5B, with additional activity against the NS3/4A serine protease [37]. Before 2011, interferon combined with ribavirin was the standard of care (SOC) for treating HCV infection [106]. The subsequent development of direct-acting antiviral agents (DAAs) for HCV reduced the cure time for infected patients to 3 months or even less [37].

Favipiravir, a nucleobase analog, was developed as an influenza antiviral drug in 2014 [89,90]. Due to its proven efficacy against influenza and existing human licensing, it was used in West Africa in 2014 to treat Ebola virus and other RNA virus infections [89], as well as for emergency treatment or palliative care of Lassa fever, norovirus, and rabies [89].

Approved in 2013, sofosbuvir (SOF) is a nucleoside inhibitor of HCV and the first effective treatment that does not require interferon [98]. Sofosbuvir has an average binding energy of −7.46 ± 0.5 kcal/mol to SARS-CoV-2 RdRp, compared with −7.28 ± 0.35 kcal/mol for HCV RdRp [100]. Sofosbuvir also suppresses DENV1 replicons in human liver Huh7 cells [29] and ZIKV RdRp [101], indicating broad-spectrum activity against multiple viruses.

Dasabuvir is the only FDA-approved non-nucleoside RdRp inhibitor [93,98] for treating HCV infection [93,98] and is also a potential broad-spectrum RdRp inhibitor [95,107,108]. It is mainly metabolized by cytochrome P450 (CYP) 2C8, with a minor contribution from CYP3A [92,97], which can terminate RNA synthesis [96]. However, resistance develops if it is not used together with other DAAs [92,94]. A major limitation of dasabuvir is its low conservation at the RdRp binding site, which limits its broad-spectrum activity [91].

Remdesivir is an adenosine analogue that was initially developed for Ebola virus treatment [109]. It subsequently became the first drug approved for the intravenous treatment of COVID-19 in 2020 [103]. After entering cells, it is converted into remdesivir triphosphate (RTP) [51], which competes with endogenous NTPs [103] and incorporates at the first base pair of the nascent RNA chain to terminate the elongation [20]. Remdesivir can also effectively inhibit RdRp activity in multiple flaviviruses, including ZIKV and DENV [104].

Originally developed as an influenza treatment, molnupiravir was ultimately approved in 2021 as a COVID-19 drug. Molnupiravir is a cytosine analogue whose triphosphate form (MTP) is incorporated by SARS-CoV-2 RdRp into the negative-sense genomic RNA (-gRNA), resulting in mutations in the viral genome [105]. However, molnupiravir may have a higher tendency to induce viral mutations than other nucleoside inhibitors.

### 4.2. Other RdRp Inhibitors

MDL-001 (pipendoxifene) is a non-nucleoside RdRp inhibitor targeting the conserved Thumb-1 site of RdRp [110]. In preclinical studies, MDL-001 was as effective as remdesivir against SARS-CoV-2 in a mouse model. It also showed favorable lung accumulation and an excellent safety profile in rats and mice [111]. It also exhibits in vitro activity against coronaviruses, mouse-adapted (MA) variants, HCV, and norovirus (NoV) [111].

Suramin, an approved antiparasitic drug, has been identified as a non-nucleoside inhibitor of SARS-CoV-2 RdRp. Suramin binds to two critical sites in the catalytic cavity of SARS-CoV-2 RdRp, and its inhibitory activity against RdRp is at least 20 times stronger than that of remdesivir [112]. Molecular docking also confirms higher binding affinity to RdRp than remdesivir [32]. It serves as a representative example of drug repurposing for RdRp inhibition, despite not being approved for clinical use.

OP4 is a structurally constrained cyclic heptapeptide with good water solubility and transmembrane ability, enabling efficient intracellular accumulation without delivery vectors. Still in the early research stage, its antiviral potency has been verified in vitro. Molecular docking further suggests broad activity against West Nile virus, Japanese encephalitis virus, ZIKV, HCV, norovirus, and coxsackievirus B3 [32,84].

Additionally, certain natural small molecules also exhibit potential RdRp inhibitory activity. It was found that fucoxanthin isolated from *Sargassum siliquastrum* can influence the levels of infectious viral particles and mRNA in ZIKV-infected cells. Molecular docking revealed a binding energy of −290.919 kcal/mol to RdRp [113]. Studies have shown that the marine natural product Harzianopyridone (HAR) can inhibit ZIKV replication by directly targeting NS5 RdRp, with an EC_50_ of 0.46 to 2.63 µM [99]. Computational methods were used to evaluate the efficacy of 10 plant chemicals in RdRp binding. Covalent docking showed compounds 7–9 bound irreversibly to the key residue CYS813 in the RdRp palm domain [114]. Molecular docking of papaya lead compounds showed seven candidates (β-sitosterol, carpaine, violaxanthin, rutin, β-carotene, pseudocarpaine, Δ7-avenasterol) had favorable binding energies to ZIKV NS5 MTase and RdRp domains, indicating their potential as viral replication inhibitors [115].

## 5. Discussion and Outlook

Positive-strand RNA viruses such as SARS-CoV-2, DENV, HCV, and CHIKV exhibit rapid mutation rates, but specific and effective antiviral drugs remain scarce [9,116,117,118]. In HCV, combined S282T and K74R mutations maintain normal viral replication while inducing SOF resistance [119]. In SARS-CoV-2, the combination of S759A and V792I confers up to 38-fold resistance to remdesivir, which is conserved in betacoronaviruses and verified in murine hepatitis virus (MHV) [120]. To overcome and delay the emergence of drug resistance, multiple innovative strategies have been proposed. Sameh S.M.’s team designed a series of dual inhibitors with two scaffolds. Based on nucleoside analogues targeting RdRp, they added a guanidine scaffold to mimic the substrate of transmembrane serine protease 2 (TMPRSS2) [83]. In another strategy inspired by peptide nucleic acid (PNA) therapeutics, researchers designed an RdRp-specific PNA antisense oligomer (PNA-CPP-1) conjugated with a cell-penetrating peptide (CPP). This agent can simultaneously bind the active site of RdRp and RNA-specific targets to inhibit viral replication [6]. At the same time, strategies including combination therapy [121], covalent inhibitor design [122], and inhibitors targeting novel allosteric sites on nsp12 [123] can also help reduce drug resistance mutations. Besides directly targeting RdRp, indirectly influencing RdRp function is also a promising research direction. Studies have shown that cyclin-dependent kinase 2 (CDK2) can phosphorylate RdRp at T20, promoting its complex formation with nsp7 and nsp8. It has been demonstrated that CDK2 inhibitors can effectively suppress SARS-CoV-2 infection [124]. Through library screening, a series of polyphenols were identified to inhibit SARS-CoV-2 and DENV RdRp activity by chelating two Mg^2+^ ions at the RdRp active site, thereby suppressing viral replication function [125]. Moreover, Li et al. (2026) [126] discovered novel covalent allosteric inhibitors of SARS-CoV-2 RdRp. The compound targets the Cys114 site on the nsp8 subunit and blocks RdRp activity.

Nucleoside inhibitors offer broader spectrum advantages due to their ability to incorporate into RNA chains [37]. However, this feature also makes them a major cause of reproductive toxicity, contributing to the difficulties in their preclinical and clinical development [22,37,59]. Therefore, developing non-nucleoside inhibitors, especially those like oligopeptides that are more host-cell friendly, offers advantages in terms of high safety and easier drug development. In addition, drug modifications like introducing bulky groups can help drugs bind better to RdRp while acting less on human polymerase II [127].

Despite the wide application of cell-free RdRp assay systems in antiviral drug screening, their limitations must be considered relative to cell-based assay systems. As cell-free assays lack the complete intracellular microenvironment, they cannot fully simulate the complex processes of viral replication, protein-protein interactions, and RNA maturation in living cells, which may lead to inconsistent results between in vitro and in vivo antiviral efficacy. In contrast, cell-based assays can reflect real physiological conditions on antiviral efficacy, but suffer from complex backgrounds, interfering factors, and difficulty in directly quantifying RdRp activity. Notably, the two systems are not mutually exclusive and can be used in a complementary manner. Most studies first validate candidate inhibitors in cell-free assays, then confirm antiviral activity and evaluate cytotoxicity in cell-based assays [24,73,128,129]. In future research, the combination of cell-free assays with biologically relevant cell-based assays will become increasingly common and essential, as this integrated strategy enables efficient, accurate, and physiologically relevant screening of RdRp inhibitors.

Developing broad-spectrum antiviral drugs targeting RdRp holds significant practical importance. It not only provides effective ways to fight viruses that have already endangered public health, but also establishes a reliable drug basis and reference for unknown viral infections [130]. Naturally, different types of drugs have different advantages and limitations. This paper aims to provide support and reference for RdRp-targeted drug research, thereby discovering novel development strategies and methodologies.

## Figures and Tables

**Table 1:** Comparative reports on the RdRp variation and conservation.

RdRp Name	Homology Percentage	Amino Acid Comparison	3D Structure (RMSD)	Comparison Range	Reference
ZIKV NS5	68% gene homology with JEV; 66% gene homology with DENV3		0.63 Å (compared to JEV)	Comparison within the genus	[11]
ZIKV primer rings	76% gene homology with DENV2 primer rings 81% gene homology with DENV3 primer rings	Priming loop residues (785–810)	1.17 Å and 1.19 Å	Comparison within the genus	[27]
16 coronaviruses	High RdRp similarity among the 16 coronaviruses	Motifs A–G		Comparison within the genus and family	[28]
DENV1 NS5	High homology to DENV2–4; Partial homology to HCV	D534, D663, D664		Comparison within the family	[29,30]
Cross-Family Viruses			A common structural core consisting of 231 residues was identified	Comparison within the different genus and family	[31]
*Coronaviridae*, *Flaviviridae*, *Picornaviridae*, *Caliciviridae*	Highly conserved amino acid residues at the active sites	Motifs A–G		Comparison within the different genus and family	[17]
SARS-CoV-2 nsp12			0.5–0.6 Å (compared to Omicron)	Comparison within the species	[32]
Picornaviruses (CVB3, HRV14, PV, EMCV, FMDV) 3D^pol^			All exhibit a typical “right-hand” conformation and share extremely high structural homology	Comparison within the family	[33]
Enterovirus C (EV–C) 3D^pol^	In recombinant group RNA > 83%; AA > 94%			Comparison within the genus	[34]
Alphaviruses (RRV, GETV, SINV, CHIKV, ONNV, VEEV, BFV, SPDV) nsP4	The amino acid sequences of the core catalytic motifs are highly conserved	Motifs A, B, C, E	1.38 Å (palm domain of RRV and SINV)	Comparison within the genus	[35]
CHIKV nsP4			1.070 Å (CHIKV–DENV), 1.248 Å (CHIKV–ZIKV), and 1.106 Å (ZIKV–DENV)	Comparison across families	[36]

**Table 2:** Comparison of the cell-based RdRp activity assay in different articles.

Viruses	Cell Line	Reporter Gene	Viral Genomic Fragment	RdRp Subunit Composition	Detection Methods	Reference
SARS-CoV-2	HEK293T A549	Gluc	SARS-CoV-2 5′ UTR and 3′ UTR	nsp12/nsp7/nsp8 gene of SARS-CoV-2	RT-qPCR; Gluc activity assay	[59]
	HEK293T	NLuc and Fluc	SARS-CoV-2 5′ UTR and 3′ UTR	nsp12/nsp7/nsp8 gene of SARS-CoV-2	Luciferase activity; Immunofluorescence Staining Assay; Z-factor calculation	[55]
	Vero cells stably expressing eGFP	mCherry	Full-length PEDV genome		mCherry fluorescent intensity	[28]
	HEK293T Vero E6	RLuc and Fluc	SARS-CoV-2 5′ UTR and 3′ UTR	nsp12/nsp7/nsp8/nsp5 gene of SARS-CoV-2	Luciferase activity; RT-PCR; Z-factor calculation	[56]
	HEK293T A549	FLuc	SARS-CoV-2 5′ UTR and 3′ UTR	nsp12 gene of SARS-CoV-2	Luciferase activity; Z-factor calculation	[57]
DENV	Huh-7	RLuc and Fluc	DENV ΔC truncated UTR	NS5 gene of DENV	Luciferase activity; RT-qPCR	[60]
ZIKV	HEK293T	Gluc	ZIKV 5′ UTR, 3′UTR and capsid 1–38 amino acids	NS5 gene of ZIKV	Luciferase activity; RT-qPCR; Z’-factor calculation	[61]
CHIKV	U-2 OS	eGFP	CHIKV 5′ UTR, 3′ UTR and subgenomic (SG) promoter	nsP1–4 gene of CHIKV	eGFP fluorescence intensity measurement; Western blot; RT-qPCR	[62]
MERS-CoV	HEK293T	FLuc and Nluc	MERS 5′ UTR and 3′ UTR	nsp12 gene of MERS-CoV	Luciferase activity; Western blot; Z-factor calculation	[63]

**Table 3:** Comparison of the cell-free RdRp activity assay in different articles.

Virus	Template	Primer	RdRp Expression System	RdRp Subunit Composition	Reporter	Detection Methods	Reference
SARS-CoV-2	Biotin-labeled short duplex RNA; self-priming long poly-U RNA	yes	Sf9 (baculovirus); *E. coli*	nsp12/nsp7/nsp8 gene of SARS-CoV-2	5′-biotin on RNA; QuantiFluor dsRNA fluorescent dye	Urea-PAGE LightShift™ EMSA Optimization; Control Kit	[58]
	An annealed RNA consisting of a primer and a template	yes	*E. coli* (BL21/Rosetta/CodonPlus)	nsp12/nsp7/nsp8 gene of SARS-CoV-2	^32^P radioactive label on 5′-end of primer RNA	Electrophoretic mobility shift assay; Urea-PAGE	[64,65]
	The 13 + 23 nt RNA template-product duplexs with a 5′-FAM fluorescent label	yes	*E. coli* (BL21/Rosetta)	nsp12/nsp7/nsp8 gene of SARS-CoV-2 (1:2:2)	5′-FAM	Urea-PAGE	[66]
	An annealed primer-template (PT) and self-priming hairpin (HP) RNA template	yes	*E. Coli* (BL21/NEB Express)	nsp12/nsp7/nsp8 gene of SARS-CoV-2 (1:3:3)	Cy5 (PT primer); 6-FAM (HP RNA)	Urea-PAGE	[67]
	T33-8/T33-1 with 6 bp hairpin	yes	*E. coli* BL21	nsp12/nsp7/nsp8 gene of SARS-CoV-2 (1:2:2)	RNA stained directly	Urea-PAGE	[68]
SARS-CoV	A template with 3′ U10 stretch	yes	*E. coli* (C2523/BL21)	nsp12	α-^32^P labeled NTP	Electrophoretic mobility shift assay; Urea-PAGE	[50]
ZIKV	A subgenomic ZIKV RNA template	no	*E. coli* BL21 (DE3) RIL	NS5	α-^32^P labeled NTP	Urea-PAGE	[11]
	An RNA template consisting of 3′UTR sequence and a poly (A) region	no	Prokaryotic expression	NS5	BBT-ATP	Measurement of the fluorescent BBT	[46]
DENV	A poly(rC) RNA template	no	*E. coli* BL21 (DE3) pLysS	NS5	^3^H-labeled GTP	Radionucleotide (3H-GMP) incorporation monitored by filter binding and liquid scintillation counting	[45]
	A hairpin-containig RNA with a 10 bp stem and a 16-nt 5′-overhang region	yes	*E. coli* KRX	NS5 and NS3	Cy5 fluorescent dye	Urea-PAGE	[69]
	single-stranded RNA poly (C) template	no	*E. coli*	NS5	PicoGreen fluorescent dye	PicoGreen Quantitation Assay	[70]
WNV	Single-stranded RNA poly (U)	no	*E. coli* BL21 (DE3)-pRIL	NS5	α-^32^P labeled NTP and SYTO-9 dye	fluorescence-based assay, Urea-PAGE	[71]
HNV	Single-stranded RNA poly (C)	no	*E. coli* BL21	HNV RdRp	PicoGreen fluorescent dye	PicoGreen Quantitation Assay	[72]
EV-A71	Single-stranded RNA poly (A)	no	*E. coli* BL21	3D^pol^	SYTO 9 fluorescent nucleic acid dye	fluorescence-based assay	[73]
CHIKV	Hairpin-structured T1 RNA; T2 ds-containing RNA	no	*E. coli* Rosetta-2 T1R (nsP4); Expi293 (nsP1)	nsP4/nsP1/nsP2	RNA stained directly	fluorescence-based assay, Urea-PAGE	[26]
HEV	Single-stranded RNA poly (C)	no	*E. coli* Lemo21	HEV-RdRp	PicoGreen fluorescent dye	PicoGreen Quantitation Assay	[74]

**Table 4:** Chemical nature of RdRp inhibitors.

Compound	Target Virus	Nucleoside Analogue or Not	Reference
Ribavirin	HCV	Guanosine analogues (synthetic)	[87,88]
Favipiravir	Influenza	Guanosine analogues	[89,90]
Dasabuvir	HCV	Non-nucleoside analogues	[91,92,93,94,95,96,97,98]
Sofosbuvir	HCV	Uridine analogues	[24,29,98,99,100,101]
Remdesivir	SARS-CoV-2, Ebola virus	Adenosine analogues	[20,44,102,103,104]
Molnupiravir	SARS-CoV-2	Cytosine analogues	[105]

## Data Availability

No new data were created or analyzed in this study. Data sharing is not applicable to this article.
